# Histopathology of melanocytic lesions in a family with an inherited BAP1 mutation

**DOI:** 10.1111/cup.12625

**Published:** 2015-10-12

**Authors:** Sally J. O'Shea, Angana Mitra, Jennifer L. Graham, Ruth Charlton, Julian Adlard, Will Merchant, Julia A. Newton‐Bishop

**Affiliations:** ^1^ Leeds Institute of Cancer and PathologySection of Epidemiology and BiostatisticsUniversity of LeedsLeedsUK; ^2^ DermatologySt. James's University HospitalLeedsUK; ^3^ HistopathologySt. James's University HospitalLeedsUK; ^4^ Clinical Genetics, Yorkshire Regional DNA LaboratorySt. James's University HospitalLeedsUK; ^5^ The Yorkshire Regional Genetics ServiceChapel Allerton HospitalLeedsUK

**Keywords:** histopathology, melanocytic lesions, melanoma

*To the Editor*,

We read with interest the report by Marusic et al.^1^ who expanded on the histopathological features of melanocytic neoplasms in a family with an inherited *BAP1* mutation.

Marusic et al.^1^ described the presence of nuclear pseudoinclusions and multinucleated melanocytes within five of six nevi from this family. The lesions were mainly intradermal, composed of large epithelioid melanocytes, and showed loss of *BAP1* expression by immunohistochemistry.

**Figure 1:**
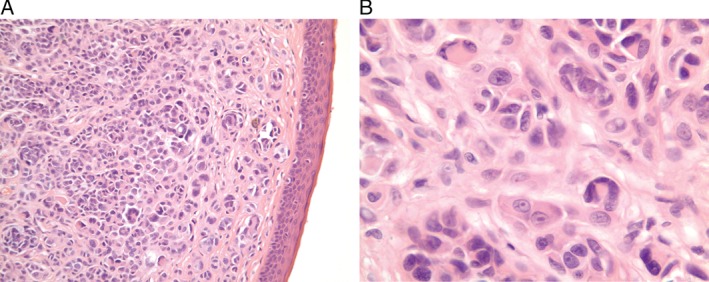
A) Histopathological findings (×20) show a predominantly intradermal melanocytic lesion. Multinucleated melanocytes can be clearly seen. B) High‐power view (×63) reveals a melanocytic lesion composed of multinucleated melanocytes. Nuclear pseudoinclusions are seen in some melanocytes. This lesion was considered to be a benign melanocytic nevus and resembled nevi described in other patients with a BAP1 mutation.

We report similar histopathological findings in melanocytic lesions excised from members of an English family in which a germline pathogenic *BAP1* mutation has been identified. The proband was diagnosed with melanoma at 26 years of age that was clinically bland and pink in appearance. This was initially considered to be a melanocytic lesion of uncertain malignant potential (MELTUMP) but on further assessment, showed significant cytological atypia and lacked maturation and was therefore treated as a melanoma. The Breslow thickness was 2.4 mm and there was no evidence of ulceration. This patient later developed a melanoma *in situ*. He had multiple pink, bland nevi one of which subsequently changed and this was excised in view of his significant history. Histopathological examination showed an intradermal melanocytic neoplasm composed of epithelioid melanocytes with abundant cytoplasm, some of which contained multinucleated cells (Fig. 1A) and nuclear pseudoinclusions (Fig. 1B).

The proband's maternal grandfather had occupational exposure to asbestos and had died of mesothelioma. His maternal uncle had a history of stage IIA melanoma, which later metastasized to a regional lymph node. Striking pleomorphism, multinucleated melanocytes and nuclear pseudoinclusions were readily identified in this nodal metastasis.

The proband's maternal aunt, presented at 44  years of age with a history of a recalcitrant scalp lesion, which had been curetted twice elsewhere in the belief that this was a cyst. The curettings had not been sent for histopathological assessment. Following a further recurrence, approximately 18  months after onset, the lesion was partially excised by general surgeons. Histopathology revealed a malignant blue nevus‐like melanoma showing a highly cellular melanocytic tumor, at least 11 mm in thickness; composed of spindle cells and some dendritic‐like melanocytes. Nuclear pseudoinclusions were noted, although distributed more sparsely within this lesion. To our knowledge, there is only one other reported case of a 64‐year‐old female with a *BAP1* germline mutation who had a malignant blue nevus‐like melanoma of the scalp.^2^ Our patient was also found to have a meningioma on computed tomography (CT) scanning.

**Figure 2:**
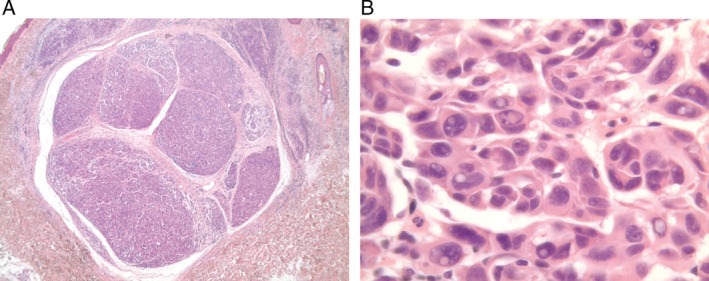
A) Low‐power view (×2.5) shows an intradermal melanocytic neoplasm composed of a nodule flanked by a more diffuse melanocytic lesion. B) High‐power view (×60) demonstrates remarkable nuclear pseudoinclusions.

Following an invitation for screening, the proband's maternal cousin had a pink lesion excised, due to a history of change. This was diagnosed as a spitzoid tumor of uncertain malignant potential (STUMP). The tumor consisted of a lobulated, intradermal nodule composed of pleomorphic epithelioid melanocytes, flanked by a more diffuse melanocytic lesion with hyperchromatic nuclei (Fig. 2A). Nuclear pseudoinclusions were numerous within the nodular component (Fig. 2B).

Atypical spitzoid tumors are known to occur in patients who have an inherited *BAP1* mutation, the histopathology of which is usually quite striking in stark contrast to the bland clinical appearance of these lesions.^3^ Recognizing this pattern of clinical and histopathological presentation is key to identifying possible carriers of a *BAP1* mutation, who have a cancer susceptibility syndrome, having a particular diathesis to cutaneous and uveal melanomas,^3^ meningiomas,^4^ mesotheliomas^5^ and renal cell cancers.^1^ Nuclear pseudoinclusions can be identified in several melanocytic tumors, including benign melanocytic nevi and even some melanomas.^1^ Although non‐specific, they appear to be a prominent feature of melanocytic lesions from patients with a germline *BAP1* mutation.

*Sally J*. *O'Shea, MB BCh BAO BMedSc^1^*
*Angana*   *Mitra, MB ChB MD^2^*
*Jennifer L*. *Graham, BDS^3^*
*Ruth* *Charlton, BA PhD^4^*
*Julian* *Adlard, MB BS^5^*
*Will* *Merchant, MB ChB FRCPath DipRCPath (Dermpath)^3^*
*Julia A*. *Newton‐Bishop, MB ChB MD FMedSci^1^*
^1^Leeds Institute of Cancer and Pathology, Section of Epidemiology and Biostatistics,
University of Leeds,
Leeds, UK.
^2^Dermatology,
St. James's University Hospital,
Leeds, UK.
^3^Histopathology,
St. James's University Hospital,
Leeds, UK.
^4^Clinical Genetics, Yorkshire Regional DNA Laboratory,
St. James's University Hospital,
Leeds, UK.
^5^The Yorkshire Regional Genetics Service,
Chapel Allerton Hospital,
Leeds, UK.
e‐mail: S.J.O'Shea@leeds.ac.uk
